# Responses of *Micropterus salmoides* under Ammonia Stress and the Effects of a Potential Ammonia Antidote

**DOI:** 10.3390/ani13030397

**Published:** 2023-01-24

**Authors:** Zhenlu Wang, Xingchen Guo, Jiao Tu, Xuan Shi, Lei Gan, Muzi Zhang, Haibo Jiang, Xiaoxue Zhang, Jian Shao

**Affiliations:** 1 Key Laboratory of Animal Genetics, Breeding and Reproduction in the Plateau Mountainous Region, Ministry of Education, Guiyang 550025, China; 2 College of Animal Science, Guizhou University, Guiyang 550025, China

**Keywords:** *Micropterus salmoides*, ammonia stress, N-carbamylglutamate, antioxidant, anti-inflammatory

## Abstract

**Simple Summary:**

Largemouth bass (*Micropterus salmoides*) has been widely cultured in China, and ammonia stress has become one of the important reasons limiting the development of largemouth bass’ farming. However, the response of largemouth bass to ammonia stress is still to be investigated. In addition, N-carbamylglutamate has been considered as a potential anti-stress additive in terrestrial animals. Currently, it remains to be explored whether N-carbamylglutamate can enhance fish resistance under environmental stress. Our findings in this study could enrich our understanding of the responses in largemouth bass under ammonia stress and help us solve the existing problem of environmental stress in largemouth bass culture.

**Abstract:**

Ammonia is a common environmental limiting factor in aquaculture. To investigate the effects of ammonia stress and explore the protective effect of N-carbamylglutamate (NCG) on *Micropterus salmoides* (*M. salmoides*), tissue sections and parameters related to oxidative stress and the inflammatory response in *M. salmoides* were carried out during the ammonia stress test and feeding test. The results demonstrated that the LC50 for 24 h, 48 h, 72 h, and 96 h under ammonia stress in *M. salmoides* were 25.78 mg/L, 24.40 mg/L, 21.90 mg/L, and 19.61 mg/L, respectively. Under ammonia stress, the structures of the tissues were damaged, and the GSH content decreased, while the MDA content increased with the increase in stress time and ammonia concentration. The NO content fluctuated significantly after the ammonia nitrogen stress. In the 15-day feeding test, with the increased NCG addition amount and feeding time, the GSH content increased while the MDA and NO contents decreased gradually in the NCG addition groups (NL group: 150 mg/kg; NM group: 450 mg/kg; NH group: 750 mg/kg) when compared with their control group (CK group: 0 mg/kg). In the ammonia toxicology test after feeding, the damage to each tissue was alleviated in the NL, NM, and NH groups, and the contents of GSH, MDA, and NO in most tissues of the NH group were significantly different from those in the CK group. The results suggested that ammonia stress caused tissue damage in *M. salmoides*, provoking oxidative stress and inflammatory response. The addition of NCG to the feed enhances the anti-ammonia ability of *M. salmoides*. Moreover, the gill and liver might be the target organs of ammonia toxicity, and the brain and kidney might be the primary sites where NCG exerts its effects. Our findings could help us to find feasible ways to solve the existing problem of environmental stress in *M. salmoides* culture.

## 1. Introduction

*Micropterus salmoides* (*M. salmoides*), also known as largemouth bass, is native to the United States and was introduced to China in 1983 [1]. Because of its strong disease resistance, rapid growth, and wide range of temperature adaptation, it has been widely cultured in China with an annual production of more than 600,000 tons (China Fisheries Statistical Yearbook, 2021). With the expansion of the culture scale, high-density culture has become the main culture mode.

Environmental stress is an important factor restricting fish culture, which can affect the health and growth rate of fish, and even cause fish mortality. The large accumulation of feed residues and animal excreta in the intensive culture mode is very likely to result in an increase in ammonia nitrogen content in culture water. Ammonia stress has become one of the important reasons limiting the development of largemouth bass farming [2]. Previous studies have demonstrated that ammonia exposure causes tissue damage and reduces the immunity of *Megalobrama amblycephala* [3]. *Poecilia reticulate* exposed to ammonia for 48 h might have been affected by its behavior, oxidative stress, and inflammatory response [4]. Histological damage to the liver and disruption of the antioxidant defense system could appear in *Trachinotus ovatus* after 96 h of ammonia stress [5]. Besides fish, ammonia stress also affects the tissue structure and physiological functions of crustaceans and bivalves [6,7]. In addition, ammonia stress may act in conjunction with other environmental factors to further affect the health of aquatic organisms [8,9]. *M. salmoides* is highly productive in aquaculture, but its physiological response to ammonia stress remains to be investigated.

In order to mitigate the adverse effects of ammonia stress on aquatic animals, some studies have been carried out to find additives against ammonia stress. There have been reports that extracts of medicinal plants, such as *Yucca schidigera*, *Moringa oleifera* leaf, and *Plantago ovata* seed, can mitigate the adverse effects of ammonia stress on aquatic animals [10,11,12]. Tea tree oil and *Clostridium butyricum* were suggested to enhance the antioxidant capacity of aquatic animals under ammonia stress [13,14]. N-carbamylglutamate (NCG) is a stable and edible active substance with the molecular formula C_6_H_10_N_2_O_5_. Its chemical structure is similar to that of arginine (N-acetylglutamate, NAG), which is one of the essential amino acids in fish and participates in the regulation of many physiological processes [15,16]. Compared with NAG, NCG has the advantages of lower cost, easier absorption, and more stable metabolism in vivo. Several studies have confirmed that NCG plays a beneficial role in the antioxidant stress in terrestrial animals, and was thus considered as a potential anti-stress additive [17,18]. However, studies on the effects of NCG on aquatic animals are still in their infancy. Currently, it remains to be explored whether NCG can enhance fish resistance under environmental stress.

In this study, the physiological responses of the gill, brain, liver, and kidney in *M. salmoides* under ammonia stress were detected, and the effects of NCG on *M. salmoides*’ resistance under ammonia stress were investigated. These results could enrich our understanding of the responses in *M. salmoides* under ammonia stress and help us to solve the existing problem of environmental stress in *M. salmoides* culture.

## 2. Materials and Methods

### 2.1. Ethical Statement

All studies were authorized by the animal care and use committee and were carried out in accordance with the Guizhou University’s Guidelines for Experimental Animals.

### 2.2. Experimental Fish and Culture Conditions

**Figure 1:**
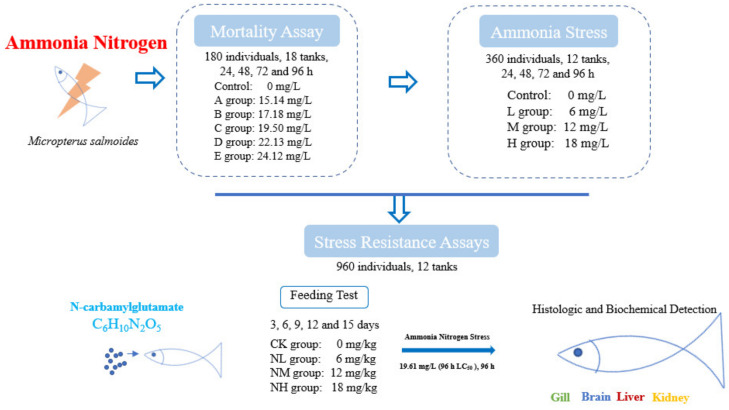
Overview of treatment and sample collection in this study.

The juvenile *M. salmoides* used in the experiment was purchased from a commercial farm (Guangdong, China) and was reared in the culture pond for one month before the experiment. During the temporary culture period, the water temperature was 22.2 ± 1.5 °C, the pH value was 7.80 ± 0.02, the dissolved oxygen > 7.5 mg/L, and the ammonia nitrogen concentration < 0.02 mg/L. The largemouth bass were fed twice daily at 5% of fish body weight with commercial feed (Guangdong Wanghai Feed, Guangzhou, China). Healthy and uniform-sized individuals (7.52 ± 0.55 cm) were randomly selected for the following test (Figure 1).

### 2.3. Cumulative Mortality Assay

Ammonia nitrogen concentration was adjusted with NH_4_Cl (analytically pure). First, a pilot test was conducted to determine the maximum tolerable concentration (15 mg/L) and the minimal lethal concentration (25 mg/L) of ammonia nitrogen in 96 h for *M. salmoides*. Then, five ammonia stress groups were set based on the pre-tests (A: 15.14 mg/L, B: 17.18 mg/L, C: 19.50 mg/L, D: 22.13 mg/L, and E: 25.12 mg/L). In addition, the group without additional ammonia nitrogen (the ammonia nitrogen concentration < 0.02 mg/L) was taken as the control group. A total of 180 fish (7.52 ± 0.55 cm) and 18 tanks were used for this test. That is, each group had three tanks, and 10 randomly selected fish were placed in each tank (with 20 L of water).

During this test, the water temperature was 22 ± 0.2 °C, the pH value was 7.80 ± 0.02, the dissolved oxygen > 6 mg/L and these fish were not fed during the tests. The ammonia nitrogen content in the water was measured every 6 h by the Nessler reagent method. The water was half exchanged every 24 h and NH_4_Cl was added to keep the ammonia nitrogen concentration stable in each group. To determine the median lethal concentration (LC50), the mortality of *M. salmoides* was recorded at 24, 48, 72, and 96 h.

### 2.4. Ammonia Stress Assays

Except for the control group, according to the 96 h LC50 in Section 2.3, three ammonia stress groups were set as follows: a low concentration group (6 mg/L, L group), a medium concentration group (12 mg/L, M group), and a high concentration group (18 mg/L, H group). Each group was set up with three tanks, and 30 fish were put into each tank. Three fish were sampled randomly from each tank at 24 h, 48 h, 72 h, and 96 h under ammonia stress for the biochemical indexes. Three fish were randomly sampled from each tank at 96 h for histological examination. The water quality parameters during the test were consistent with those in Section 2.2.

### 2.5. Stress Resistance Assays

#### 2.5.1. Effect of NCG on *M. salmoides*

NCG was selected as an anti-stress agent, and the NCG addition groups were set at a 0 mg/kg (CK group), 150 mg/kg (NL group), 450 mg/kg (NM group), and 750 mg/kg (NH group) according to a previous study [19]. A total of 960 fish (13.35 ± 0.55 cm) and 12 tanks (400 L) were used for this test. That is, each group had three tanks, and 80 randomly selected fish were placed in each tank. The fish were fed twice a day (8:00 and 16:00) under satiety feeding conditions, and leftover feed would be retrieved after 1 h of feeding. The water temperature was 22 ± 1 °C, the pH value was 7.80 ± 0.02, the dissolved oxygen >7 mg/L, and the ammonia concentration was < 0.02 mg/L. This feeding test lasted for 15 days. Three fish were sampled from each tank at 3, 6, 9, 12, and 15 days for subsequent index detection.

The feed was provided by Wanghai Feed (Guangdong, China), and the main raw material composition was fish meal, soybean meal, flour, fish oil, vitamins, and minerals. The nutrient ratios were as follows: crude protein ≥ 48.0%, crude fat ≥ 2.0%, crude fiber ≤ 5.0%, crude ash ≤ 15.0%, lysine ≥ 3.0%, moisture ≤ 12.0%, and total phosphorus between 0.5 and 3.0%.

#### 2.5.2. Effect of NCG on Ammonia Nitrogen Resistance

After the feeding test, an ammonia nitrogen tolerance test was carried out for these NCG addition groups. Specifically, 30 individuals were randomly selected from the remaining fish in each tank and put into a new tank with 100 L water. The 96 h LC50 (19.61 mg/L) was used as the stress concentration of ammonia nitrogen to conduct an ammonia nitrogen resistance test. Three fish were randomly sampled from each tank every 24 h for the detection of biochemical index. At 96 h after stress, three fish were randomly sampled from each tank for histological analysis. Other water quality parameters during the test were consistent with those in the feeding test.

### 2.6. Index Detection

#### 2.6.1. Sample Collecting

In our present study, for sampling in Section 2.3, Ammonia stress assays, and Section 2.4, Stress resistance assays, three fish were randomly taken from each tank at each sampling point. After anesthesia with MS-222, the gill, brain, liver, and kidney of *M. salmoides* were placed on ice.

For the samples used for histological analysis, each tissue was fixed with 4% paraformaldehyde solution for further tissue sectioning. For the samples used for biochemical indexes analysis, 50~100 mg of each tissue were taken and stored at −20 °C for further enzyme activity determination.

#### 2.6.2. Histological Detection

After a series of steps, including fixation, dehydration, embedding, sectioning, and staining, the tissues were observed by a microscope (Nikon, Japan), and then photographed and analyzed with CaseViewer 2.4 software.

#### 2.6.3. Biochemical Detection

After obtaining the sample weight of gill, brain, liver, and kidney, the 10% tissue homogenates were prepared according to the mass (g):volume (mL) = 1:9 by adding normal saline. After centrifuging at 500 rpm/min at 4 °C for 10 min, the supernatants were collected for the biochemical index assay. The assays were performed according to the manufacturer’s instructions using glutathione (GSH), malondialdehyde (MDA), and nitric oxide (NO) kits, and the total protein content in each tissue was measured using a total protein (TP) assay kit for the calculation of the above indexes. All the above kits were purchased from Nanjing Jiancheng Bioengineering Institute.

### 2.7. Statistical Analysis

In this study, data were analyzed by SPSS 22.0 for one-way ANOVA based on the Tukey test. A *p* value < 0.05 was considered a significant difference. Drawings were completed by Origin 2021, and the data are presented as the mean ± SD of triplicate measurements.

## 3. Results

### 3.1. Analysis of Mortality

In this study, no mortality occurred in the control group during the 96 h of ammonia stress. In the treatment groups, the mortality of juvenile *M. salmoides* gradually increased with the increase in concentration and time. More specifically, in the A group, the fish showed mortality at 96 h under stress. In the B group, the fish showed mortality at 48 h. In the C, D, and E groups, the fish showed mortality at 24 h under stress, and the cumulative mortality rate in E group was 96.67% at 96 h under stress.

The LC50 values were calculated by Probit analysis, and the results showed that the LC50 for 24 h, 48 h, 72 h, and 96 h under ammonia stress were 25.78 mg/L, 24.40 mg/L, 21.90 mg/L, and 19.61 mg/L, respectively. The safe mass concentration was calculated using the following formula: Safe concentration (SC) = LC50 (96 h) × 0.01, and the results showed that the SC of ammonia stress for juvenile *M. salmoides* was 0.20 mg/L (Table 1).

### 3.2. Histological Analysis under Ammonia Stress

**Figure 2:**
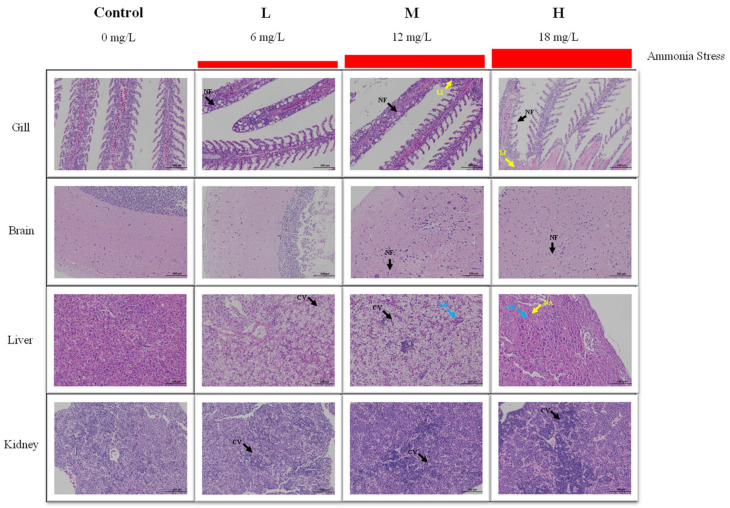
Effect of ammonia stress on histology of juvenile *M. salmoides* at 96 h. NF: nuclear fragmentation; LI: lymphocyte infiltration; CV: cytoplasmic vacuolization; EB: eosinophilic bodies; NA: nucleolar aggregation.

After 96 h of ammonia stress, the structural changes of the tissues of *M. salmoides* are shown in Figure 2. For the gill, the gill structure in the control group was clear and regular, and the gill filament was complete. A mild cell necrosis and nuclear fragmentation were visible in the L group. Cell necrosis, nuclear fragmentation, and lymphocyte infiltration were observed in the M and H groups.

For the brain, the cerebral neurons of the control and L groups were closely arranged, and the morphological structure of neurons was regular. In the M group, a part of the neuronal cytoplasmic vacuolization was observed. In the H group, a large amount of neuronal necrosis and vacuolization of the cytoplasm were noticed.

For the liver, there was no obvious damage in the control group. In the L group, many hepatocytes were vacuolized in cytoplasm. In the M group, cytoplasmic vacuolization and eosinophilic bodies were found. In the H group, hepatocytes swelled, and eosinophils, and hepatocyte nucleolar aggregation were observed.

For the kidney, the morphology and structure of the cells were regular in the control group. In the L and M groups, mild cell apoptosis and nuclear fragmentation were observed. More apoptosis and nuclear fragmentation were found in the H group.

### 3.3. Biochemical Analysis under Ammonia Stress

#### 3.3.1. GSH Content

**Figure 3:**
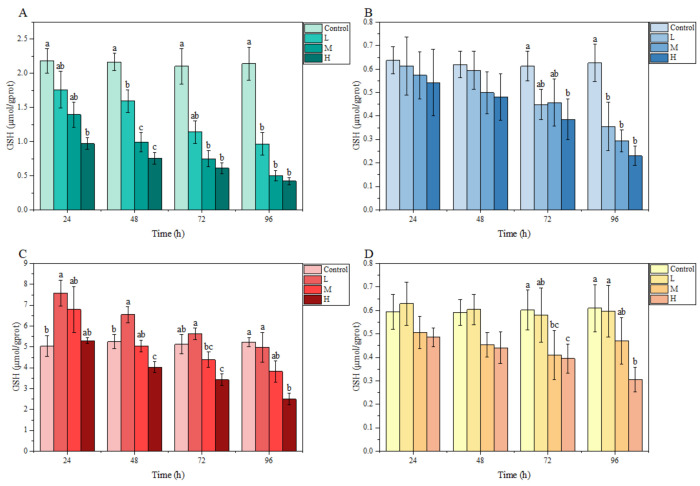
Effects of ammonia stress on GSH content of juvenile *M. salmoides.* (**A**–**D**) represent the GSH content in the gill, brain, liver, and kidney, respectively. The bars represent the mean ± S.D. (*n* = 3). Different letters indicate statistical differences (*p* < 0.05).

Under ammonia stress, in general, the GSH content in each tissue of *M. salmoides* in each treatment group showed a decreasing trend with the increase in stress time and concentration. Specifically, there was a significant difference in GSH content in the gill and liver compared with the control group at 24 h after ammonia stress. Among them, the GSH content of the H group in the gill was significantly lower than that of the control group, but the GSH content of L group in liver was significantly higher than that of the control group. At 72 h, the GSH content of the H group in the brain, as well as the M and H groups in the kidney, were significantly lower than that in the control group. At 96 h, the GSH content of L and M groups in gill and brain were significantly lower than that of the control group, and the GSH content of H group in all tissues of this study was significantly lower than that of the control group (Figure 3).

#### 3.3.2. MDA Content

**Figure 4:**
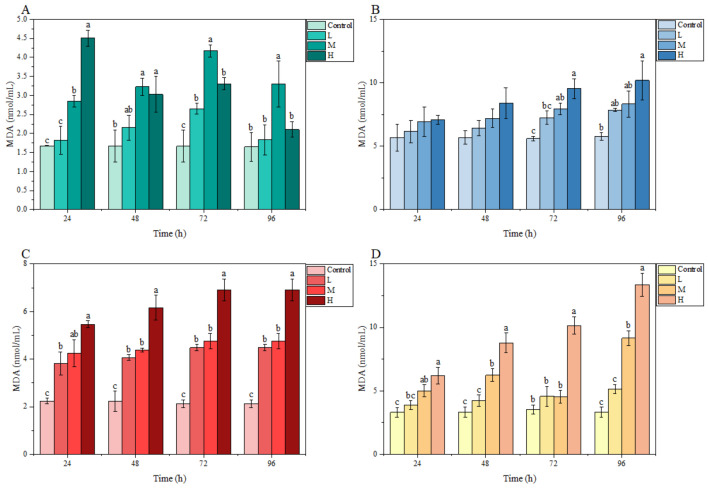
Effects of ammonia stress on MDA content of juvenile *M. salmoides.* (**A**–**D**) represent the MDA content in the gill, brain, liver, and kidney, respectively. The bars represent the mean ± S.D. (*n* = 3). Different letters indicate statistical differences (*p* < 0.05).

In the gill, the MDA content fluctuated under ammonia stress, and peaked at 72, 72, and 24 h in the L, M, and H groups, respectively. In the brain, liver, and kidney, MDA contents in each treatment group overall showed an increasing trend with stress time and ammonia nitrogen concentration. In particular, compared with the control group, a significant difference was observed in the MDA content in the liver and kidney at 24 h and in these three tissues at 72 h. At 96 h, the MDA content in the liver of the L group, in the liver and kidney of the M group, as well as in the brain, liver, and kidney of the H group, were significantly higher than those of the control group (Figure 4).

#### 3.3.3. NO Content

**Figure 5:**
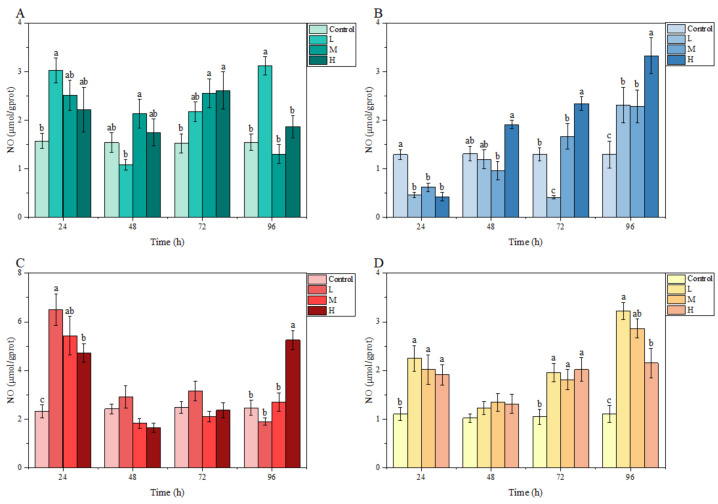
Effects of ammonia stress on NO content of juvenile *M. salmoides.* (**A**–**D**) represent the NO content in the gill, brain, liver, and kidney, respectively. The bars represent the mean ± S.D. (*n* = 3). Different letters indicate statistical differences (*p* < 0.05).

Under ammonia stress, an obvious fluctuation was found in the NO content of each tissue of *M. salmoides* in each treatment group relative to the control group. At 24 h, compared with the control group, the NO content in each treatment group was higher in the gill, liver, and kidney, but significantly lower in the brain. At 48 h, no significant differences were observed in any tissue from each treatment group relative to the control group. At 96 h, the NO content in the gill, brain, and kidney of the L group, the brain and kidney of the M group; and the brain, liver, and kidney of the H group were significantly higher than those of the control group (Figure 5).

### 3.4. Analysis of NCG Impact in Feeding Test

**Figure 6:**
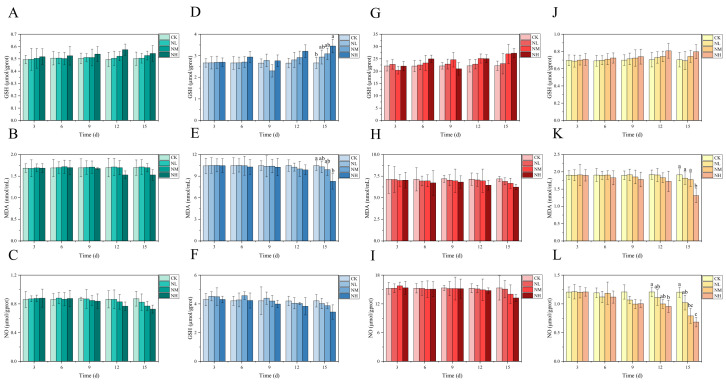
Effects of dietary supplementation of NCG on juvenile *M. salmoides.* (**A**–**C**, **D**–**F**, **G**–**I**, and **J**–**L**) represent the biochemical parameters (GSH, MDA, and NO) in the gill, brain, liver, and kidney. The bars represent the mean ± S.D. (*n* = 3). Different letters indicate statistical differences (*p* < 0.05).

Overall, in the 15 days feeding test, the GSH content in each tissue of *M. salmoides* in each treatment group increased gradually with the increase in NCG-added concentration and feeding time when compared with that in the control group; however, the change trend of MDA and NO content was opposite to that of GSH content. Of these, the NO content in the kidney was significantly lower in the NH group than in its control group at day 12. At day 15, NO content in the kidney was significantly lower in the NM group than in its control group. In the NH group, GSH content in the brain was significantly higher, whereas MDA and NO contents were significantly lower than those of the control group (Figure 6).

### 3.5. Analysis of NCG Impact on Ammonia Nitrogen Resistance

#### 3.5.1. Effects of NCG on Tissue Structure under Ammonia Stress

**Figure 7:**
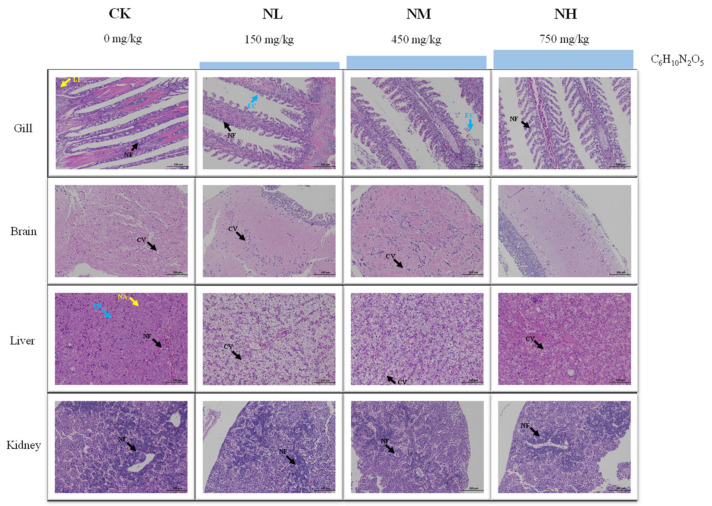
Effect of NCG on histology of juvenile *M. salmoides* under ammonia stress. NF: nuclear fragmentation; LI: lymphocyte infiltration; EC: epithelial cells; CV: cytoplasmic vacuolization; EB: eosinophilic bodies; NA: nucleolar aggregation.

The change in ammonia resistance of *M. salmoides* was investigated after feeding a diet containing NCG for 15 days. According to HE staining, the degree of damage each tissue in each group accumulated was different after 96 h of ammonia stress (Figure 7).

To be specific, in the gill, cell necrosis, nuclear fragmentation, and lymphocyte infiltration were found in the CK group. Nuclear fragmentation and exfoliated epithelial cells were visible in the NL and NM groups. Mild cell necrosis and nuclear fragmentation were observed in the NH group. In the brain, cytoplasmic vacuolization was found in CK group, NL group, and NM group. Meanwhile, the cell showed a normal image and no obvious tissue damage was found in the NH group. In the liver, eosinophilic bodies, nucleolar aggregation, and nuclear fragmentation were found in the CK group. In the NL group and NM group, vacuolization of hepatocyte cytoplasm was observed, and in the NH group, there was a mild cytoplasmic vacuolization. In the kidney, a large number of apoptosis and nuclear fragmentation were observed in the CK and NL groups. The NM and NH groups showed mild to moderate apoptosis and nuclear fragmentation.

#### 3.5.2. Effects of NCG on Biochemical Indexes under Ammonia Stress

**Figure 8:**
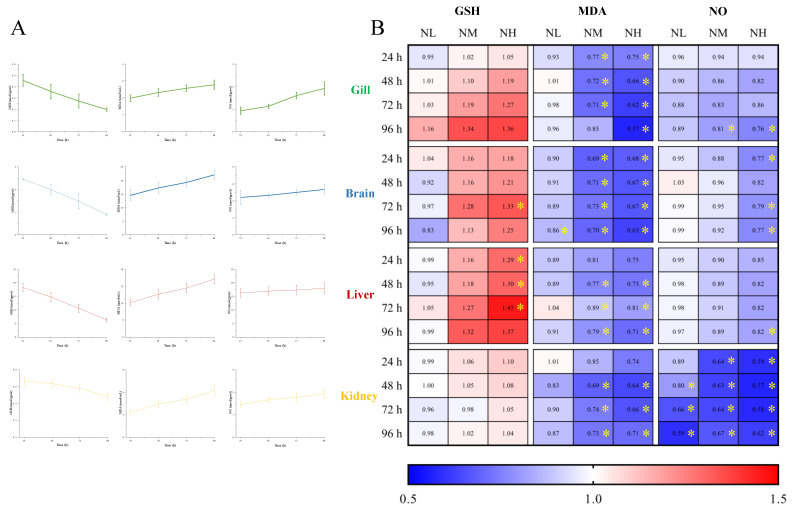
Effects of NCG on biochemical indexes of juvenile *M. salmoides* under ammonia stress. (**A**) The changes of biochemical parameters (GSH, MDA, and NO) in the gill, brain, liver, and kidney of *M. salmoides* under ammonia stress. The bars represent the mean ± S.D. (*n* = 3). (**B**) The heat map of the fold change of biochemical parameters between the NCG added groups and the CK group * indicate statistical difference (*p* < 0.05).

To investigate the effect of NCG on ammonia nitrogen resistance of *M. salmoides*, the contents of GSH, MDA and NO in each tissue of *M. salmoides* under ammonia stress were detected. The trends of the above biochemical indicators in the gill, brain, liver and kidney in the CK group were similar to those in the H group of 3.3, ammonia stress (Figure 8A).

The fold change of each index in the NL, NM, and NH groups relative to the CK group is shown in Figure 8B. The results demonstrated that after 15 days of NCG feeding, the GSH content of *M. salmoides* under ammonia stress was higher than that of the CK group, and the MDA and NO contents were lower than that of the CK group. The difference of each index between NCG adding group and its CK group, in absolute terms, was positively correlated with the amount of NCG added to the diet.

Among them, the GSH content of the NH group in the brain at 72 h, and in the liver at 24, 48, and 72 h were significantly higher than those in the CK group. For MDA content, compared with the CK group, it was significantly lower at 96 h of the brain in the NL group, as well as in the NM and NH groups at most sampling points. For NO content, most of the significant differences were observed in the kidney, and the NO content of the NH group at 96 h was significantly lower than that of the control group in all tissues in this study.

## 4. Discussion

*M. salmoides* is currently one of the most important cultured fish species in China. However, ammonia stress imposes serious challenges to their survival and growth in its intensive aquaculture model. This disadvantage has limited the further development of *M. salmoides* farming. It is necessary to explore the response of *M. salmoides* under ammonia stress and discover the potential stress resistant agent.

### 4.1. Effects of Ammonia Stress on Mortality and Histology

Ammonia nitrogen is the most common environmental pollutant in intensive aquaculture and can adversely affect aquatic organisms such as fish, bivalves and crustaceans [2]. Yan et al. showed that the LC_50_ for 96 h under ammonia stress in juvenile hybrid grouper (♀ *Epinephelus fuscoguttatus* × ♂ *E. lanceolatu*) was 25.0 mg/L [20]. Liu et al. conducted an acute ammonia nitrogen toxicity test on juvenile golden pompano (*Trachinotus ovatus*) and found that the LC_50_ (96 h) for it was 26.9 mg/L [5]. Hao et al. reported that the LC_50_ for 96 h under ammonia stress in juvenile *Carassius auratus* was 135.4 mg/L [21]. In the present study, the 96 h LC_50_ for juvenile *M. salmoides* under ammonia stress was 19.61 mg/L. This result suggested that the tolerance of juvenile *M. salmoides* to ammonia is at a low level among these reported fish.

Studies have demonstrated that ammonia usually enters the organism through the gills and epidermis of fish, causing damage to gill tissues and leading to respiratory distress in fish [22,23,24]. After ammonia diffusion into the tissues, it could damage organs in the fish, inhibit their growth or even lead to their death [3,6,25,26]. Through histological analysis of this study, we found that under ammonia stress, especially at the H group, the gill, brain, liver, and kidney of *M. salmoides* had serious tissue damage, including massive cell necrosis, nuclear fragmentation, and cytoplasmic vacuolation. The severity of damage in various tissues of *M. salmoides* is proportional to the ammonia concentration in the water.

### 4.2. Effect of NCG on Stress Tolerance on M. salmoides

Oxidative stress is one of the important response mechanisms of aquatic animals to environmental stress, and several studies have shown that ammonia stress can trigger oxidative stress in fish [27,28,29]. GSH, which is essential for maintaining cellular redox homeostasis and regulating cellular metabolism, is an effective antioxidant in response to environmental stress [30]. MDA is an important product of membrane lipid peroxidation following free radical attack on biofilms, and the changes in its content can reflect the damage degree of the biofilm system [31]. GSH and MDA are considered to be reliable and sensitive biological indicators for assessing the antioxidant capacity and the extent of oxidative stress damage in fish after environmental stress, respectively [21,26]. In addition, the inflammatory response is one of the mechanisms by which the organism responds to external stimuli, and NO is involved in a variety of physiological processes, including the inflammatory response [32,33]. Under normal conditions, endogenous NO has a role in suppressing inflammatory responses. Many drugs could exert their therapeutic effects by inhibiting the production of NO [34,35].

In this study, the addition of NCG in feed caused an increase in GSH content as well as a decrease in MDA and NO content in *M. salmoides* during the feeding test, and the degree of these changes was proportional to the feeding time and NCG content. This result indicated that NCG contributed to improving the antioxidant and anti-inflammatory ability of *M. salmoides*. In the subsequent stress resistance test, the GSH content in all tissues of *M. salmoides* in the NCG-added group was higher than that of the CK group, and the content of MDA and NO was lower than that of the CK group, the degree of change was also positively proportional to the NCG content. This result implied that NCG could enhance the tolerance of *M. salmoides* under ammonia stress. Several studies have confirmed that the addition of NCG to feeds could help terrestrial animals to improve growth and reproductive performance, alleviate histological damage as well as improve energy status [36,37,38]. Huang et al. found that moderate amounts of NCG could improve lipid metabolism and reduce inflammation in *Lateolabrax japonicus* organisms and its cells cultured in vitro [19,39]. Combined with the above results, we suggested that NCG is a potential anti-stress additive, and it may work by enhancing antioxidant and anti-inflammatory capacity and reducing oxidative damage in fish. However, the optimal amount of the NCG addition and more detailed anti-stress mechanisms need to be further explored.

### 4.3. Tissue Specific Responses to Ammonia Stress and NCG Addition

Fish organs, such as the gill, liver, and kidney, play an important role in the defense against environmental toxicity. The gill is the first organ of fish to respond to harmful environmental conditions [40,41]. The liver and kidney are involved in the detoxification of the organism and are therefore a target organ for ammonia toxicity [42,43,44]. In addition, studies have confirmed that ammonia can also damage the central nervous system in fish [45]. In addition, histological data suggest that fish have a larger cranial space in the skull compared to mammals and can withstand greater cranial pressure [26]. In this study, the tissue section results demonstrated that there was no obvious damage in the brain of *M. salmoides* in the control and L groups. Therefore, relative to other tissues, the brain of fish may be less sensitive to ammonia stress.

**Figure 9:**
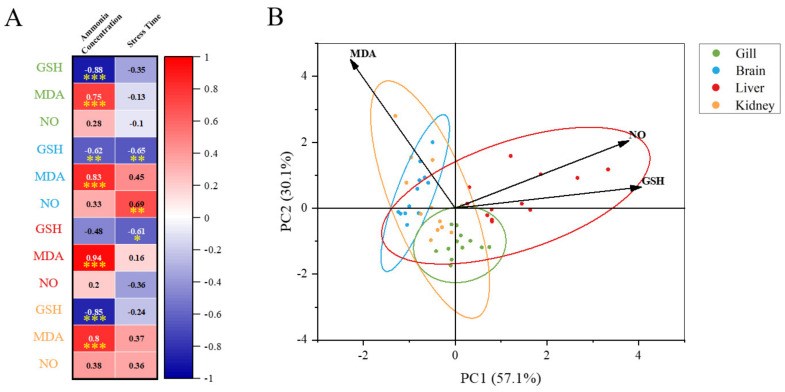
Multi−parameter integration analysis. (**A**) The Pearson’s correlation heat map of biochemical parameters (GSH, MDA, and NO). Asterisks (* *p* < 0.05; ** *p* < 0.01; *** *p* < 0.001) indicate significant difference between the two groups; (**B**) principal component analysis (PCA) plots for biochemical parameters in different tissues under ammonia stress.

By comparing the changes of each tissue under ammonia stress in our present study (Figure 9), we found that the response of *M. salmoides* is different in the gill, brain, and liver, and kidney. Pearson’s correlation analysis demonstrated that the most significant correlation occurred in the change of MDA content and ammonia concentration in the liver. The correlation between the GSH content and ammonia concentration in the gill was the most significant among the negative correlations. This result indicated that the gill and liver of *M. salmoides*, especially the liver, were more sensitive to ammonia stress compared to other tissues. Moreover, the principal component analysis (PCA) was used for comparative analysis among tissues, demonstrating a clear separation between the gill and brain. It has been demonstrated that *Nile tilapia*, *Megalobrama amblycephala*, and *Ctenopharyngodon idellus* also show the tissue-specific response in response to ammonia stress, and our results were consistent with the above findings [3,9,46].

What is more, in this study, the significant changes in GSH, MDA, and NO contents only occurred in the brain and kidney of *M. salmoides* during 15 days of the feeding test. This result suggested that the brain and kidney were more sensitive to NCG when compared with other tissues and might be the target organs for NCG. Based on the above results, we can conclude that the regulation of antioxidant and anti-inflammatory systems under ammonia stress, as well as the effect of NCG in *M. salmoides*, were tissue-specific.

## 5. Conclusions

In summary, ammonia stress caused tissue damage to the gill, brain, liver, and kidney in *M. salmoides*. The antioxidant and anti-inflammatory responses would be activated under ammonia stress, and NCG could enhance the anti-ammonia ability of *M. salmoides*. Moreover, the gill and liver were more sensitive to ammonia stress compared to other tissues, and the brain and kidney might be the target organs for NCG. These results may provide new clues for finding feasible ways to solve the existing problem of ammonia stress in *M. salmoides* culture.

## Figures and Tables

**Table 1:** The cumulative mortality assay under ammonia stress on juvenile *M. salmoides*.

Group	Ammonia Concentration (mg/L)	Cumulative Number of Deaths (Individual)			
		24 h	48 h	72 h	96 h
Control	0	0	0	0	0
A	15.14	0	0	0	1
B	17.18	0	1	7	10
C	19.50	2	8	11	16
D	22.13	8	12	16	20
E	25.12	11	14	21	29
LC50 (mg/L)		25.78	24.40	21.90	19.61

## Data Availability

The data presented in this study are available on request from the corresponding author.

## References

[1] Bai J., Lutz-Carrillo D.J., Quan Y., Liang S. (2008). Taxonomic status and genetic diversity of cultured largemouth bass *Micropterus salmoides* in China. Aquaculture.

[2] Xu Z., Cao J., Qin X., Qiu W., Mei J., Xie J. (2021). Toxic effects on bioaccumulation, hematological parameters, oxidative stress, immune responses and tissue structure in fish exposed to ammonia nitrogen: A review. Animals.

[3] Guo H., Chen S., Ouyang K., Kuang Y., Yang H., Wang Y., Tang R., Zhang X., Li D., Li L. (2022). Evaluation of ammonia nitrogen exposure in immune defenses present on spleen and head-kidney of Wuchang bream (*Megalobrama amblycephala*). Int. J. Mol. Sci..

[4] Zhang C., Ma J., Qi Q., Xu M., Xu R. (2023). Effects of ammonia exposure on anxiety behavior, oxidative stress and inflammation in guppy (*Poecilia reticulate*). Comp. Biochem. Physiol. Part C Toxicol. Pharmacol..

[5] Liu M., Guo H., Zhu K., Liu B., Liu B., Guo L., Zhang N., Yang J., Jiang S., Zhang D. (2021). Effects of acute ammonia exposure and recovery on the antioxidant response and expression of genes in the Nrf2-keap1 signaling pathway in the juvenile golden pompano (*Trachinotus ovatus*). Aquat Toxicol..

[6] Lu J., Yao T., Shi S., Ye L. (2022). Effects of acute ammonia nitrogen exposure on metabolic and immunological responses in the Hong Kong oyster *Crassostrea hongkongensis*. Ecotoxicol. Environ. Saf..

[7] Zhuo H., Liu J. (2022). Nuclear Factor Interleukin 3 (NFIL3) Participates in regulation of the NF-ΚB-mediated inflammation and antioxidant system in *Litopenaeus vannamei* under ammonia-n stress. Fish Shellfish Immunol..

[8] Kuang Y., Guo H., Ouyang K., Wang X., Li D., Li L. (2023). Nano-TiO_2_ aggravates immunotoxic effects of chronic ammonia stress in zebrafish (*Danio rerio*) intestine. Comp Biochem Physiol C Toxicol Pharmacol.

[9] Esam F., Khalafalla M.M., Gewaily M.S., Abdo S., Hassan A.M., Dawood M.A.O. (2022). Acute ammonia exposure combined with heat stress impaired the histological features of gills and liver tissues and the expression responses of immune and antioxidative related genes in Nile tilapia. Ecotoxicol. Environ. Saf..

[10] Elbialy Z.I., Salah A.S., Elsheshtawy A., Rizk M., Abualreesh M.H., Abdel-Daim M.M., Salem S.M.R., Askary A.E., Assar D.H. (2021). Exploring the multimodal role of *Yucca schidigera* extract in protection against *Chronic ammonia* exposure targeting: Growth, metabolic, stress and inflammatory responses in Nile tilapia (*Oreochromis niloticus* L.). Animals.

[11] Kaleo I.V., Gao Q., Liu B., Sun C., Zhou Q., Zhang H., Shan F., Xiong Z., Bo L., Song C. (2019). Effects of *Moringa oleifera* leaf extract on growth performance, physiological and immune response, and related immune gene expression of *Macrobrachium rosenbergii* with vibrio anguillarum and ammonia stress. Fish Shellfish Immunol..

[12] Ahmadifar E., Kalhor N., Yousefi M., Adineh H., Moghadam M.S., Sheikhzadeh N., Moonmanee T., Hoseinifar S.H., Van Doan H. (2022). Effects of dietary *Plantago ovata* seed extract administration on growth performance and immune function of common carp (*Cyprinus carpio*) fingerling exposed to ammonia toxicity. Vet. Res. Commun..

[13] Liu M., Gao Q., Sun C., Liu B., Liu X., Zhou Q., Zheng X., Xu P., Liu B. (2022). Effects of dietary tea tree oil on the growth, physiological and non-specific immunity response in the giant freshwater prawn (*Macrobrachium rosenbergii*) under high ammonia stress. Fish Shellfish Immunol..

[14] Sun C., Tadese D.A., Wangari M.R., Zhou Q., Zheng X., Liu B., Tamiru M., Dagne A., Janssens G.P.J., Zhao Y. (2022). Amelioration of ammonia-induced intestinal oxidative stress by dietary *Clostridium butyricum* in giant freshwater prawn (*Macrobrachium rosenbergii*). Fish Shellfish Immunol..

[15] Liang H., Mokrani A., Ji K., Ge X., Ren M., Pan L., Sun A. (2018). Effects of dietary arginine on intestinal antioxidant status and immunity involved in Nrf2 and NF-ΚB signaling pathway in juvenile blunt snout bream, *Megalobrama amblycephala*. Fish Shellfish Immunol..

[16] Mommsen T.P. (2001). Paradigms of growth in fish. Comp. Biochem. Physiol. B Biochem. Mol. Biol..

[17] Xiao L., Cao W., Liu G., Fang T., Wu X., Jia G., Chen X., Zhao H., Wang J., Wu C. (2016). Arginine, n-carbamylglutamate, and glutamine exert protective effects against oxidative stress in rat intestine. Anim. Nutr..

[18] Zhang H., Jin Y., Wang M., Loor J.J., Wang H. (2020). N-carbamylglutamate and l-arginine supplementation improve hepatic antioxidant status in intrauterine growth-retarded suckling lambs. RSC Adv..

[19] Huang H.Y., Chen P., Liang X.F., Wu X.F., Wang C.P., Gu X., Xue M. (2019). Dietary n-carbamylglutamate (NCG) alleviates liver metabolic disease and hepatocyte apoptosis by suppressing ERK1/2-MTOR-S6K1 signal pathway via promoting endogenous arginine synthesis in Japanese seabass (*Lateolabrax japonicus*). Fish Shellfish Immunol..

[20] Yan X., Chen Y., Dong X., Tan B., Liu H., Zhang S., Chi S., Yang Q., Liu H., Yang Y. (2021). Ammonia toxicity induces oxidative stress, inflammatory response and apoptosis in hybrid grouper (♀ *Epinephelus fuscoguttatus* × ♂ *E. lanceolatu*). Front. Mar. Sci..

[21] Hao M., Zuo Q., Zhang W., Feng Y., Wang L., Yu L., Zhang X., Li J., Huang Z. (2019). Toxicological assessment of ammonia exposure on *Carassius auratus* red var. living in landscape waters. Bull. Environ. Contam Toxicol..

[22] Benli A.C.K., Köksal G., Ozkul A. (2008). Sublethal ammonia exposure of Nile tilapia (*Oreochromis niloticus* L.): Effects on gill, liver and kidney histology. Chemosphere.

[23] Cong M., Wu H., Cao T., Ji C., Lv J. (2019). Effects of ammonia nitrogen on gill mitochondria in clam *Ruditapes philippinarum*. Environ. Toxicol. Pharmacol..

[24] Rodrigues R.V., Schwarz M.H., Delbos B.C., Carvalho E.L., Romano L.A., Sampaio L.A. (2011). Acute exposure of juvenile cobia *Rachycentron canadum* to nitrate induces gill, esophageal and brain damage. Aquaculture.

[25] Wang S., Li X., Zhang M., Jiang H., Wang R., Qian Y., Li M. (2021). Ammonia stress disrupts intestinal microbial community and amino acid metabolism of juvenile yellow catfish (*Pelteobagrus fulvidraco*). Ecotoxicol. Environ. Saf..

[26] Zhang W., Xia S., Zhu J., Miao L., Ren M., Lin Y., Ge X., Sun S. (2019). Growth performance, physiological response and histology changes of juvenile blunt snout bream, *Megalobrama amblycephala* exposed to chronic ammonia. Aquaculture.

[27] Cheng C., Yang F., Ling R., Liao S., Miao Y., Ye C., Wang A. (2015). Effects of ammonia exposure on apoptosis, oxidative stress and immune response in pufferfish (*Takifugu obscurus*). Aquat Toxicol..

[28] Gao X., Fei F., Huang B., Meng X., Zhang T., Zhao K., Chen H., Xing R., Liu B. (2021). Alterations in hematological and biochemical parameters, oxidative stress, and immune response in *Takifugu rubripes* under acute ammonia exposure. Comp. Biochem. Physiol. C Toxicol. Pharmacol..

[29] Seo J.S., Haque M.N., Nam S.E., Kim B.M., Rhee J.S. (2020). Inorganic nitrogen compounds reduce immunity and induce oxidative stress in red seabream. Fish Shellfish Immunol..

[30] Xiao W., Loscalzo J. (2020). Metabolic responses to reductive stress. Antioxid Redox Signal.

[31] Gaweł S., Wardas M., Niedworok E., Wardas P. (2004). Malondialdehyde (MDA) as a lipid peroxidation marker. Wiad. Lek..

[32] Ghasemi M. (2019). Nitric Oxide: Antidepressant mechanisms and inflammation. Adv. Pharmacol..

[33] Guzik T.J., Korbut R., Adamek-Guzik T. (2003). Nitric oxide and superoxide in inflammation and immune regulation. J. Physiol. Pharmacol..

[34] Wang L., Qiu X.-M., Hao Q., Li D.-J. (2013). Anti-inflammatory effects of a Chinese herbal medicine in atherosclerosis via estrogen receptor β mediating nitric oxide production and NF-ΚB suppression in endothelial cells. Cell Death Dis..

[35] Yatoo M.I., Gopalakrishnan A., Saxena A., Parray O.R., Tufani N.A., Chakraborty S., Tiwari R., Dhama K., Iqbal H.M.N. (2018). Anti-inflammatory drugs and herbs with special emphasis on herbal medicines for countering inflammatory diseases and disorders—A review. Recent Pat Inflamm Allergy Drug Discov..

[36] Ma Y., Zhou S., Lin X., Zeng W., Mi Y., Zhang C. (2020). Effect of dietary n-carbamylglutamate on development of ovarian follicles via enhanced angiogenesis in the chicken. Poult Sci..

[37] Yang J., Zheng J., Fang X., Jiang X., Sun Y., Zhang Y. (2021). Effects of dietary n-carbamylglutamate on growth performance, apparent digestibility, nitrogen metabolism and plasma metabolites of fattening Holstein bulls. Animals.

[38] Zhang H., Peng A., Yu Y., Guo S., Wang M., Coleman D.N., Loor J.J., Wang H. (2019). N-carbamylglutamate and l-arginine promote intestinal absorption of amino acids by regulating the mTOR signaling pathway and amino acid and peptide transporters in suckling lambs with intrauterine growth restriction. J. Nutr..

[39] Huang H., Zhang X., Liang X., Wu X., Gu X., Han J., Xue M. (2021). N-carbamoylglutamate improves lipid metabolism, inflammation, and apoptosis responses in visceral adipocytes of Japanese seabass (*Lateolabrax japonicus*), in Vivo and in Vitro. Anim. Nutr..

[40] Pritchard J.B. (2003). The gill and homeostasis: Transport under stress. Am. J. Physiol. Regul. Integr. Comp. Physiol..

[41] Zhong Y., Duan Z., Su M., Lin Y., Zhang J. (2021). Inflammatory responses associated with hyposaline stress in gill epithelial cells of the spotted scat *Scatophagus argus*. Fish Shellfish Immunol..

[42] van der Oost R., Beyer J., Vermeulen N.P.E. (2003). Fish bioaccumulation and biomarkers in environmental risk assessment: A review. Environ. Toxicol. Pharmacol..

[43] Mooney T.J., Pease C., Trenfield M., van Dam R., Harford A.J. (2018). Modeling the pH-ammonia toxicity relationship for *Hydra viridissima* in soft waters with low ionic concentrations. Environ. Toxicol. Chem..

[44] Zhang W., Sun S., Ge X., Xia S., Zhu J., Miao L., Lin Y., Liang H., Pan W., Su Y. (2018). Acute effects of ammonia exposure on the plasma and haematological parameters and histological structure of the juvenile blunt snout bream, *Megalobrama amblycephala*, and post-exposure recovery. Aquac. Res..

[45] Van der Linden A., Verhoye M., Nilsson G.E. (2001). Does anoxia induce cell swelling in carp brains? In vivo MRI measurements in crucian carp and common carp. J. Neurophysiol..

[46] Zhao C., Xu J., Xu X., Wang Q., Kong Q., Xu F., Du Y. (2019). Organ-specific responses to total ammonia nitrogen stress on juvenile grass carp (*Ctenopharyngodon idellus*). Environ. Sci. Pollut. Res. Int..

